# "Sell an Ox" - The Price of Cure for Hepatitis C in Two Countries

**DOI:** 10.15171/ijhpm.2019.135

**Published:** 2019-12-11

**Authors:** Ora Paltiel, Workagegnehu Hailu, Zenahebezu Abay, Avram Mark Clarfield, Martin McKee

**Affiliations:** ^1^School of Public Health, Hadassah-Hebrew University of Jerusalem, Jerusalem, Israel.; ^2^Department of Internal Medicine, Gondar University Hospital, University of Gondar, Gondar, Ethiopia.; ^3^Medical School for International Health, Ben Gurion University, Beersheva, Israel.; ^4^Department of Public Health & Policy, London School of Hygiene and Tropical Medicine, London, UK.

**Keywords:** Hepatitis C, Ethiopia, USA, Antiviral Therapy, Out-of-Pocket Expenses

## Abstract

Chronic hepatitis C virus (HCV) infection, associated with severe liver disease and cancer, affects 70 million people worldwide. New treatments with direct-acting-antivirals offer cure for about 95% of affected individuals; however, treatment costs may be prohibitive in both the poorest and richest nations. Opting for cure may require sacrificing essential household assets. We highlight the financial dilemmas involved, drawing parallels between Ethiopia and the United States, countries where universal health coverage does not yet exist. The World Health Organization (WHO) declaration for HCV eradication by 2030 will only become reality if universal access to efficacious and affordable treatment is guaranteed for everyone.

The patient lay in the bed in the crowded emergency room of the Gondar University Hospital, in northern Ethiopia, barely attentive to the crowd of doctors surrounding him during morning rounds. The residents presented the case to their attending physician. The patient was a 40-year-old male who presented with a gradual onset of fatigue, jaundice, loss of appetite and increased abdominal girth. On physical examination, he looked malnourished, with temporal wasting, icteric sclera, and abdominal swelling with shifting dullness. His blood test showed that he was positive for hepatitis C virus (HCV) antibody and he was diagnosed with hepatic decompensation and ascites due to cirrhosis, secondary to chronic HCV infection. A plan was agreed. The medical team would tap the ascites and offer nutritional and supportive care.

The attending physician asked the house staff if they were aware of a new access program providing treatment for HCV at highly subsidized rates, offering potential to cure the disease. Did they suggest this to the patient? They replied that indeed they had mentioned it, but the patient could not afford the medication; he was a poor farmer. The attending physician asked “How many oxen does he have? If more than one, tell him to “sell an ox.”

Hepatitis C, affecting over 70 million people worldwide,^1^ has traditionally been considered a lifelong infection, associated with a high risk of cirrhosis, hepatocellular carcinoma, hepatic failure, serious extra-hepatic manifestations and premature death. Now, however, direct-acting antiviral (DAA) therapies promise elimination of the virus in almost 95% of cases.^2^ To this end, Target 3.4 of the Sustainable Development Goals aims to reduce mortality by one third from non-communicable diseases.^3^ As such global eradication of hepatitis C by 2030 is currently viewed as a realistic goal,^4,5^ but only if Target 3 of the Sustainable Development Goals, universal access to effective care can be guaranteed.

In an equitable world, all HCV-infected patients would have an equal chance to access treatment. One of the ironies of global health is the fact that many citizens of the richest and most citizens of the poorest countries in the world, are denied the chance of cure for HCV. In the case of the patient in Gondar, the ox is both a literal and figurative entity. The decision whether or not to sell an important asset, such as an ox, has relevance both literally and metaphorically far beyond the borders of Ethiopia.

The 2000 World Health Report noted that “Since the poor are condemned to live in their bodies just as the rich are, they need protection against health risks fully as much.”^6^ According to this report, one of the objectives of a health care system is “providing financial protection against costs of ill-health including the burden that a specific illness places on individuals and their families, but also the cost of care and cure.” Curative therapies may be associated with substantial charges for new drugs and technologies as well as ancillary costs. In the case of DAAs these include diagnostic testing, patient transportation to a care centre, imaging and end of therapy viral load testing to establish eradication or sustained viral response. DAAs may be genotype-specific, requiring genotypic confirmation of the HCV strain, or pan-genomic (obviating the requirement for genotyping).

Although current guidelines advocate therapy for all infected individuals, regardless of the extent of liver fibrosis,^7^ some countries have restricted access to DAA to those with advanced disease (requiring imaging to confirm fibrosis) or to those who have quit or have promised to refrain from alcohol consumption.

The price of cure for HCV varies widely across countries, with costs ranging from $300 to $84 000 per course – almost a 3000% difference .^8^ This variability stems from many sources, including disparities in patent law, availability of generics, the vagaries of domestic and international pharmaceutical pricing etc. Thus, depending on where you live, affordability of DAAs fluctuates widely. In the African context, even expanded access programs incur costs equivalent or exceeding 100%-300% of the yearly income for the poorest populations.^9^ Recently, a highly subsidized program, supported by manufacturers, has been inaugurated in Ethiopia for expanded access for treatment of HCV with a genotype-specific DAA.

A review of three months’ data from the laboratory in Gondar University Hospital found 90 blood samples positive for HCV antibody. Forty-five patients were found to be eligible for treatment and were counselled to receive treatment but only four had initiated DAA therapy, primarily because of significant financial barriers. Table outlines the costs involved, which can be considered representative for public hospitals in Ethiopia.

**Table:** Pharmaceutical and Ancillary Costs Involved in Receiving DAAs for HCV in Ethiopia

**Item**	**Price in ETB** ^a^	**US$ Equivalent**	**£ Equivalent**
Drug for 3 months	18 000	585.4	444.7
Drug for 6 months	36 000	1170.7	889.3
Non-pharmaceutical costs			
HCV antibody test	50	1.6	1.2
Liver and renal function tests	300	9.8	7.4
Complete blood count	50	1.6	7.4
Ultrasound	200	6.5	4.9
Viral load (start of treatment)	1500	48.8	37.1
Viral load (end of treatment)	1500	48.8	37.1
Genotyping	5000	162.6	123.5
Transport	300	9.8	7.4
Total non-pharmaceutical costs	8900	289.4	219.9
Price of an ox	15 000-25 000	487.8-650.4	370.6-494.1
GDP per capita 2018	25 500	829.3	629.9

Abbreviations: HCV, hepatitis C virus; GDP, gross domestic product; DAAs, direct-acting antivirals. ^a^ETB: Ethiopian Birr, exchange rates 30.7 Birr/$, 40.5 Birr/£ as at December 5, 2019. Note: Drug prices were obtained from the list of the Ethiopian Pharmaceutical Fund and Supply Agency, which purchases medicines for all public hospitals and supplies them at a subsidized cost. Costs of viral load and genotype testing are also consistent across the country as they are undertaken in India, arranged by an Ethiopian laboratory agent. Ultrasound, haematology, and clinical chemistry costs, which represent a small share of the total, are the reported national average for government hospitals and private clinics, although these costs can be higher in large cities such as Addis Ababa.

So why suggest the patient sell an ox? Eighty percent of Ethiopia’s 105 million inhabitants still live in rural areas and engage in farming. Oxen are used as draught animals and as sires of calves. But they also act traditionally as a type of insurance policy. Minimally a pastoral family requires at least one ox for survival, especially for ploughing and other field activities, but a second ox can be used as a hedge against personal calamity,^10^ This insurance may also be communal, since traditionally, when a member of village falls ill, or suffers another financial catastrophe, another villager(s) may sell an ox to underwrite the expenses of medical care for his neighbor.

Prices of livestock vary according to the season,^11^ and according to conditions elsewhere in the country and, beyond it, in neighbouring countries. The farmer can, however, expect to realise a sum that is approximately equivalent to the annual gross domestic product (GDP) per capita in Ethiopia (Table).^12^ This is a lot of money, much more than the approximately 2% of GDP that people in low- and middle-income countries report being willing to pay annually for health insurance.^13^

Patients such as the man in the hospital in Gondar have a choice. He can sell an ox to purchase treatment that will, very likely, save his life. Or he may decide that the cost is too high for him and his family and take a chance. The decision has nothing to do with clinical need. Rather, the most important factor may be whether he has one ox or two. And the farmer’s decision is far from irrational; if he has only one animal, selling it could very well lead to a downward spiral into debt, affecting him and his family, from which there is little chance of escape. He could also borrow money to finance his cure, but that again will put him in debt. According to the World Bank, approximately 9% of Ethiopians do borrow to pay for medical care; similarly 7% of rural Americans need to borrow to finance medical debt.^14^

So from Gondar, we move to the United States. Initially, HCV DAAs were priced at $60 000 to $80 000 for a 12-week course of treatment, although a newer agent glecaprevir/pibrentasvir (Mavyret; AbbVie, North Chicago, IL) has a markedly lower price tag of $26 400 for an 8-week treatment.^15^ Once again, as in Ethiopia, the decision to accept treatment in the United States is based not on clinical need but rather on the patient’s financial/insurance status. The share of the population lacking health insurance is much higher in Ethiopia than in the United States. The Ethiopian Community Based Health Insurance scheme covers only an estimated 11 million, or 10.5% of the population,^16^ while a nascent Social Health Insurance scheme covers a relative small number of workers in the formal sector, leaving about 85% of people without cover, while 27.5 million Americans, or 8.5% of the population lack insurance.^17^ However, those Americans who are uninsured, and many more whose coverage is limited, must cover this entire expense, as well as the ancillary costs of testing and transport, all out of pocket.

Even if they are insured, American patients may struggle to obtain the requisite approval for DAA treatment, ^18^ and it is not unusual for insurance companies to demand a 20% to 30% cost-sharing responsibility to be borne by the patient.^19^ Cost sharing of up to $12 000 means that the cost of cure for HCV in the United States is roughly equivalent to the price not of an ox, but that of the cheapest car, or about 20% of the GDP per capita of the United States. Inevitably, this generates inequalities and, as with the sale of an ox, the loss of a car may similarly impair a family’s ability to obtain income, especially if they live in a rural area lacking public transport.

Even among those insured by Medicare, out-of-pocket expenses for HCV treatment for those without low-income subsidies is estimated at $6297 to $10 889.^20^ Among those enrolled with Kaiser Permanente, one of the major health maintenance organisations, Hispanics, African Americans and other minorities are less likely to initiate therapy than whites, as are those with behavioral risk factors (such as drug, alcohol and tobacco misuse) as well as HIV co-infection.^21^

Yet, not surprisingly, when treatment is offered to the uninsured in financial assistance programs which cover both pharmaceutical costs and testing, compliance and treatment outcomes were found to be similar to those of insured patients.^22^

For many Americans, costs of medications and illness can be major determinants of personal bankruptcy and home foreclosures.^23^ In a recent survey of a representative sample of the US population, when asked to “choose between two treatments, identical in every way except for their probability of a cure and their risk of driving the individual into bankruptcy,” the majority (70.5%) stated that they would choose cure “at all costs,” even at risk of bankruptcy, 21% weighted cure and financial protection equally and a surprising 8.5% chose financial solvency over cure.^24^ Thus, the dilemma of selling an ox, or an ox-equivalent, is a common one in countries where universal health coverage is unavailable due to financial constraints, lack of political will, or both. Just like the man in Gondar, due to a lack of universal health care, many Americans with hepatitis C must make choices. They too face the risk of a downward spiral into debt.

In an ideal world, the promise of cure for HCV should become a reality for all its citizens. Governments, non-governmental organisations and the pharmaceutical industry have shown that they can co-operate in the treatment and control of infectious diseases. Creative solutions need to be devised to adequately reward pharmaceutical companies for their advances.^25^ Furthermore, concerted efforts similar to those made in the face of HIV-AIDS need to be made to rid the world of HCV and to ensure access to curative care for patients whether they live in Africa or America; all this without their having to compromise their homes, their livestock, their livelihoods or their lives.

## Ethical issues

Not applicable.

## Competing interests

Authors declare that they have no competing interests.

## Authors’ contributions

OP conceived the study together with AMC and MM and drafted the first draft. All authors contributed to the writing of the manuscript. WH and ZA collected data on HCV patients and expenses related to HCV care in Ethiopia. All authors read and approved the final draft.

## Authors’ affiliations

^1^School of Public Health, Hadassah-Hebrew University of Jerusalem, Jerusalem, Israel. ^2^Department of Internal Medicine, Gondar University Hospital, University of Gondar, Gondar, Ethiopia. ^3^Medical School for International Health, Ben Gurion University, Beersheva, Israel. ^4^Department of Public Health & Policy, London School of Hygiene and Tropical Medicine, London, UK.
